# Obituary – Professor Joseph Lau 1947—2024

**DOI:** 10.1097/JS9.0000000000001692

**Published:** 2024-07-17

**Authors:** R. David Rosin, Riaz Agha

**Figure:**
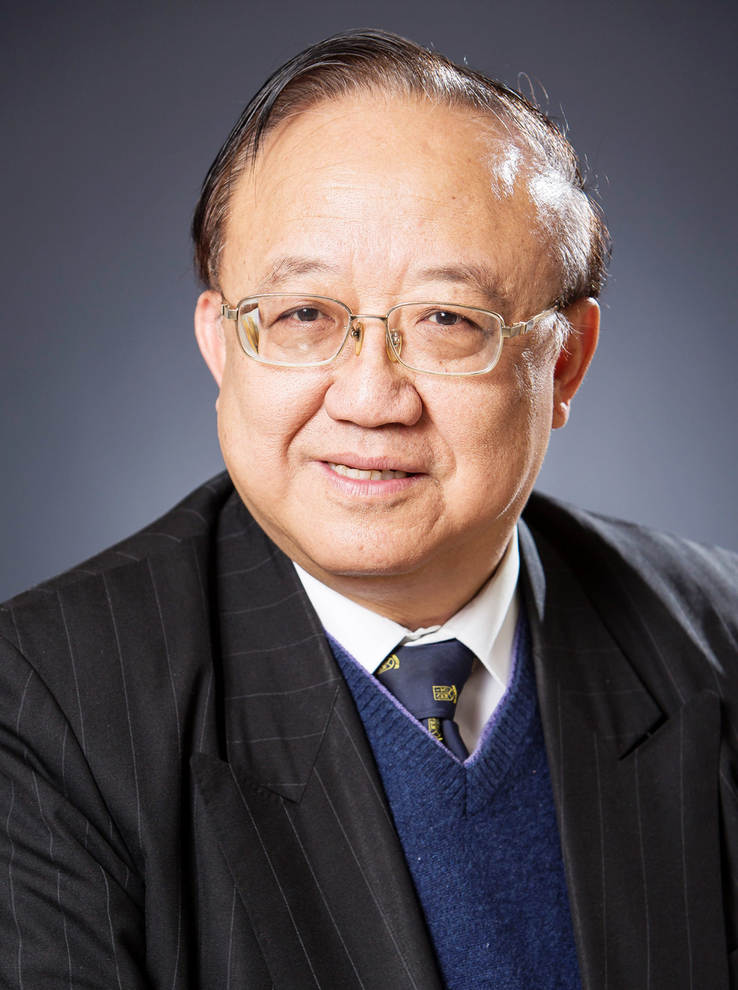


Joseph Lau was a dedicated surgeon, an excellent teacher, and a prolific researcher. He held many high positions in Hong Kong, which included being Chairman of the Medical Council, Academician of the Chinese Academy of Sciences, the founding Master of Lee Woo Sing College at the Chinese University of Hong Kong and President of the College of Surgeons of Hong Kong.

Professor Lau left a legacy of unparalleled dedication and service to the medical community of China and beyond. He was a world-renowned expert in hepato-, pancreatic-biliary surgery. The Medical Council of Hong Kong stated ‘he served Council with total commitment and passion’. His unwavering dedication to the advancement of surgical knowledge and his profound impact on the lives of countless people will forever be revered. Here we given our reflections of working with this great man.

**Prof David Rosin**

I was fortunate to meet Joseph when he worked for Professor G.B. Ong at Queen Mary Hospital in Pokfulam, Hong Kong, as a visiting Senior Registrar in 1978. At that time, he was working at Queen Elizabeth Hospital, Kowloon, but he attended all the meetings with Prof Ong’s team, and we became good friends.

When appointed as a consultant to St. Mary’s Hospital in London the following year, he came to visit on many occasions, always giving a superb lecture to the surgical staff. I would always see him and attend rounds when I visited Hong Kong.

When I was invited to become Editor-in-Chief of the *International Journal of Surgery*, I quickly asked Joseph to become an editor. He did sterling work in this position. When I relinquished the position as Editor-in-Chief in 2017, I thought immediately that Joseph would be the perfect successor. He was better than perfect and had many great new ideas, especially as to how to improve our Impact Factor. He was far better at this than I had been, attracting high-class research papers. In his time as Editor-in-Chief, the Impact Factor climbed from 3.158 to 15.3. An amazing achievement and a 384% increase!

**Dr Riaz Agha**

Joseph was an exceptional Editor and led the IJS with distinction and to great success. He helped build our brand in China and worked like a machine to stay on top of the considerable workload the position involves over his nearly 6-year stewardship as Editor-in-Chief 2017–2023. He was a great source of personal support and a great mentor to me. I was always impressed by how he attended board meetings in London every year, flying in from Hong Kong and going back the same day – I never could persuade him to stay for dinner, such was his work ethic.

The IJS will miss his wisdom, guidance, attention to detail and hard work, and we will miss a good friend and colleague.

## Ethical approval

Not applicable.

## Consent

Not applicable.

## Source of funding

Not applicable.

## Author contribution

Both helped write the article.

## Conflicts of interest disclosure

The authors declare no conflicts of interest.

## Guarantor

Riaz Agha.

## Data availability statement

Not applicable.

## Provenance and peer review

Not applicable.

